# Identification and Characterization of *Shaker* Potassium Channel Gene Family and Response to Salt and Chilling Stress in Rice

**DOI:** 10.3390/ijms25179728

**Published:** 2024-09-08

**Authors:** Quanxiang Tian, Tongyuan Yu, Mengyuan Dong, Yue Hu, Xiaoguang Chen, Yuan Xue, Yunxia Fang, Jian Zhang, Xiaoqin Zhang, Dawei Xue

**Affiliations:** 1 College of Life and Environmental Sciences, Hangzhou Normal University, Hangzhou 311121, China; quanxiang@hznu.edu.cn (Q.T.); 2022111010017@stu.hznu.edu.cn (M.D.); xg.chen@hotmail.com (X.C.); 2023111010055@stu.hznu.edu.cn (Y.X.); 20173008@hznu.edu.cn (Y.F.); 2 Zhejiang Provincial Key Laboratory for Genetic Improvement and Quality Control of Medicinal Plants, Hangzhou Normal University, Hangzhou 311121, China; 3 State Key Laboratory of Rice Biology and Breeding, China National Rice Research Institute, Hangzhou 311400, China; zhangjian@caas.cn

**Keywords:** rice, *Shaker* potassium channel, abiotic stress, bioinformatics analysis, expression patterns

## Abstract

*Shaker* potassium channel proteins are a class of voltage-gated ion channels responsible for K^+^ uptake and translocation, playing a crucial role in plant growth and salt tolerance. In this study, bioinformatic analysis was performed to identify the members within the *Shaker* gene family. Moreover, the expression patterns of rice *Shaker(OsShaker)* K^+^ channel genes were analyzed in different tissues and salt treatment by RT–qPCR. The results revealed that there were eight *OsShaker* K^+^ channel genes distributed on chromosomes 1, 2, 5, 6 and 7 in rice, and their promoters contained a variety of cis-regulatory elements, including hormone-responsive, light-responsive, and stress-responsive elements, etc. Most of the *OsShaker* K^+^ channel genes were expressed in all tissues of rice, but at different levels in different tissues. In addition, the expression of *OsShaker* K^+^ channel genes differed in the timing, organization and intensity of response to salt and chilling stress. In conclusion, our findings provide a reference for the understanding of *OsShaker* K^+^ channel genes, as well as their potential functions in response to salt and chilling stress in rice.

## 1. Introduction

Soil salinity is a global environmental problem that restricts the growth and productivity of plants [1]. The area of saline soils in the world is about 950 million hectares [2]. It is estimated that, by the middle of the 21st century, about 50% of the world’s arable land will be affected by soil salinization, and this proportion is expected to increase further due to global warming and irrational irrigation [3]. Rice is an important cereal crops for the world’s population. However, it is one of the most salt-susceptible cereals, especially at the seedling stage. Therefore, it is crucial from the perspective of global food security to identify the key genes and breed new varieties of salt-tolerant rice. 

Potassium (K^+^), which accounts for 2–10% of plant dry weight, is essential not only for normal growth and development, but also for salt stress response [4,5,6]. For instance, salt stress normally leads to K^+^ efflux accompanying the accumulation of Na^+^ in the cytoplasm, which can competitively combine the K^+^ binding sites of enzymes, as Na^+^ and K^+^ share similar chemical properties, resulting in cellular metabolic disorders. Therefore, maintaining cytoplasmic K^+^ concentration is vital for plant salt tolerance [7,8]. K^+^ exists in the soil in a variety of forms, However, due to the adsorption of silicates, the available K^+^ content in the soil is usually very low [9,10]. Moreover, plant cells need a high concentration of K^+^ to maintain normal metabolism. Cytoplasmic K^+^ content is relatively stable, ranging from 60 to 200 mM, while vacuolar K^+^ concentration is more variable, ranging from 20 to 500 mM [4,11,12]. Therefore, plants have evolved efficient K^+^ absorption and transport systems to maintain optimal growth in low potassium environments or under abiotic stresses [13].

In plants, K^+^ absorption and transport are executed mainly by K^+^ transporters and channels. There are three families of K^+^ transporters in plants [14]. HAK (high-affinity K^+^)/KUP (K^+^ uptake)/KT(K^+^ transporter) is the largest family of K^+^ transporters, only found in plants, with a K^+^/H^+^ symporter function [15]. The HKT (High-Affinity Potassium Transporter) family is mainly responsible for the transport of K^+^/Na^+^ cotransporters or Na^+^ [16]. The CPA (Cation proton antiporters) family can be further divided into the CHX (Cation/H^+^ exchangers), NHX (Na^+^/H^+^ exchangers), and KEA (K^+^ efflux antiporters) subfamilies [14], and only some members have been reported to be involved in K^+^ transport in *Arabidopsis* [17,18]. Four K^+^ channel families exist in plants: *Shaker* (*Shaker*-type K^+^ channels), TPK (Two-pore K^+^-channels), Kir (K^+^-inward rectifier)-like channels, and NSCC (non-selective cation channels). TPK members generally consists of two ion-conducting pores and four transmembrane domains, and this structure is thought to be similar to that of the KCNK (Two-pore-domain) family in animals [19]. Kir-like has only two transmembrane domains and one ion-conducting pore, similar to animal Kir. TPK and Kir-like channels cannot perceive osmolarity changes due to their lack of osmotic pressure-sensing structures. However, many TPK and Kir-like channels have an EF-hand domain at the C-terminal that can sense changes in calcium ions, suggesting that they may be sensitive to intracellular or intercellular changes in Ca^2+^ [20]. TPK family members are mainly located in the vacuole membrane to mediate K^+^ transport, and a few are localized in the plasma membrane [21]. NSCC probably plays the major role in K^+^ uptake at high concentrations (>10 mM). *Shaker* channels were the first potassium channel proteins identified and are responsible for the uptake and translocation of K^+^ [22]. The basic structure of the *Shaker* channel consists of four alpha-subunits that surround each other to form a central aqueous pore for the permeation of K^+^ [22]. A typical α-subunit usually contains a short intracellular N-terminus of only about 60 amino acids, a C-terminus, six transmembrane segments (S1–S6) and a conserved ion-conducting pore region [23]. The fourth of these transmembrane structures (S4) senses and transmits voltage changes and controls the opening and closing of the channel [23]. The *Shaker* family can be divided into three functional subfamilies based on their voltage dependence: inwardly rectifying potassium channels (Kin), weakly rectifying potassium channels (K-weak), and outwardly rectifying potassium channels (K-out) [24].

The *Shaker* family is important in plant potassium ion uptake and transport under abiotic stresses. *Shaker* family member AtGORK is an outward K^+^ channel, which negatively regulates salt tolerance in *Arabidopsis*, and lack of *AtGORK* in *Arabidopsis* could enhance salt tolerance [25]. In rice, *OsKAT1* can significantly increase the cellular K^+^ content and K^+^/Na^+^ ratio of suspension cells and alleviate the inhibitory effect of salt stress on cell proliferation [26]. In addition, it was recently found that, under salt stress, the phloem-localized *OsAKT2* is responsible for re-transporting K^+^ leaking from the phloem into the phloem and transporting it through the phloem cycle to critical sites, such as roots and young leaves, to maintain K^+^ and Na^+^ homeostasis at these sites, the mutation of *OsAKT2* resulted in significant sensitivity to salt stress in rice [27,28]. *OsK5.2* expression was significantly up-regulated in the shoot under salt stress, resulting in a large outflow of K^+^ from the stomatal guard cells, stomatal closure, and a rapid decrease in the transpiration flow, reducing the transport of Na^+^ to the above-ground part along with the transpiration flow, and improving the salt tolerance of rice [29]. In eggplant, expression of *Shaker* family genes *AKT1*, *KAT1* and *SOS1* was significantly up-regulated in leaves of the salt-tolerant variety ST118 [30]. In addition, *Shaker* family members have also been found to be involved in the regulation of salt tolerance in other plants. GmAKT1 mediates K^+^ uptake and maintains Na^+^/K^+^ homeostasis under salt stress, and overexpression of *GmAKT1* enhances salt tolerance in *Arabidopsis* and soybean [31,32]. In *Zygophyllum xanthoxylum*, AKT1 enhances salt tolerance by regulating root cell Na^+^/K^+^ homeostasis [33]. PbrKAT1 exhibits typical inward rectifier currents in *Xenopus* oocytes, and its activity is inhibited by external Na^+^ [34].

Rice is one of the most important food crops in the world, providing food for more than half of the global population. The *Shaker* family plays an important role in plant resistance to abiotic stress, However, this family has been less widely studied and lacks systematic bioinformatics analysis for identification and response to adversity stress in rice. In this study, we employed bioinformatics and publicly available data to identify the *Shaker* potassium channel in the rice genome and performed analysis of its gene structure, phylogenetic tree, conserved protein sequences, and promoter cis-acting elements, etc. In addition, we used reverse transcription quantitative polymerase chain reaction(RT–qPCR) to investigate the tissue expression pattern of the *Shaker* genes and their transcriptional response to salt and chilling stress. Our results provide a set of potential candidate *Shaker* K^+^ channel genes for the future genetic modification of K^+^ transport and salt and chilling tolerance in higher plants.

## 2. Results

### 2.1. Identification of the OsShaker K^+^ Channel Genes

**Figure 1:**
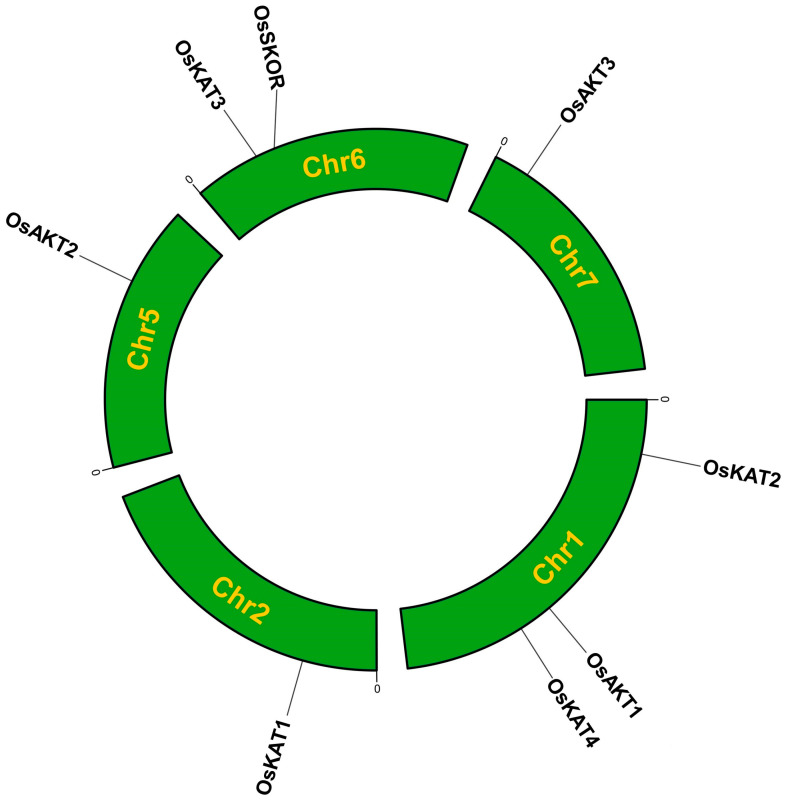
Chromosome localization analysis of *Shaker* K^+^ channel genes in rice. The green arc length represents chromosome length.

Eight *OsShaker* K^+^ channel genes were identified in the rice genome, which were distributed on chromosomes 1, 2, 5, 6, and 7 (Figure 1, Supplementary Table S1). The *OsShaker* K^+^ channel genes are named according to the homologous relationships with *Arabidopsis*.

The results of physicochemical property analysis showed that the amino acid lengths of the *Shaker* family proteins ranged from 373 to 935; the amino acid number of the OsKAT4 was the lowest, while the OsAKT1 has the longest peptide. The molecular masses of the proteins ranged from 41.67 to 102.02 kDa, with the smallest being the OsKAT4 and the largest being the OsAKT1, with 41.67 kDa and 102.02 kDa, respectively. The isoelectric points ranged from 5.66 to 10.9, with the smallest being the OsSKOR and the largest being the OsAKT3. The hydrophobicity values of the proteins ranged from −0.3 to 0.308, and the hydrophobicity values of the proteins were distributed in both positive and negative, indicating that the hydrophobicity of the rice *Shaker* family proteins is not fixed. The predicted subcellular localization showed that all *OsShaker* K^+^ channel proteins were localized in the cytoplasmic membrane. In addition, OsAKT3 and OsKAT1 have extra nuclear localization signal peptides (Table 1, Supplementary Table S2).

### 2.2. OsShaker K^+^ Channel Proteins’ Sequence Alignment, Gene Structure and Conserved Motif Analysis

**Figure 2:**
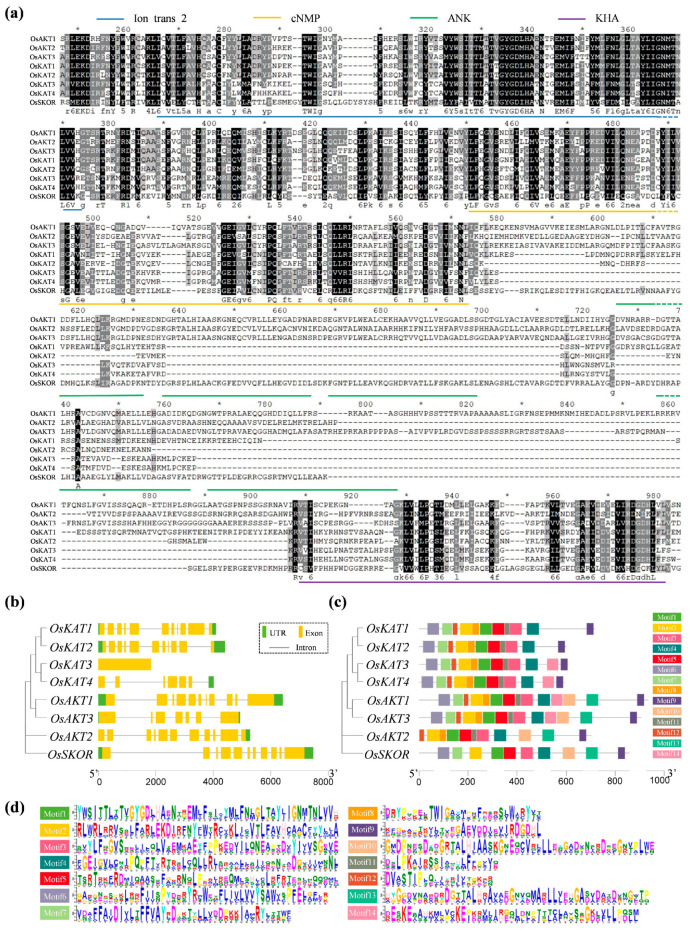
Analysis of protein sequence, gene structure and motifs of the *OsShaker* K^+^ channel proteins. (**a**) Protein sequence comparison of the *OsShaker* K^+^ channel, the underlining in different colors representing different structural domains. (**b**) Analysis of the gene structure of *OsShaker* K^+^ channel genes, containing introns, exons, and UTR region, indicated by the black line, yellow and green rectangles, respectively. (**c**) Analysis of conserved motifs of the *OsShaker* K^+^ channel proteins, different colored rectangles representing different motifs. (**d**) Sequence Logo of the *OsShaker* K^+^ channel protein motifs. Different letters represent different amino acids.

Comparative analysis of the *OsShaker* K^+^ channel protein sequences showed that they have four highly conserved domains: the Ion_trans_2 domain, the cNMP domain, the ANK domain and the KHA domain (Figure 2a). Structural analysis of the *OsShaker* K^+^ channel genes demonstrated that the length of the family genes ranged from 1776 (OsKAT3) to 7499 (OsSKOR) bp. The number of exons in the *OsShaker* K^+^ channel genes ranged from 1 to 11, except for *OsKAT3*, which contained one exon; the other members contained more than six exons, among which *OsAKT1*, *OsAKT2*, *OsKAT1*, *OsKAT2*, and *OsSKOR* contained 11 exons (Figure 2b).

Conserved motif analysis is important for exploring the structural composition of proteins, as well as their function. The MEME online tool was used to analyze the number and distribution of the Motifs of *OsShaker* K^+^ channel proteins; a total of 14 Motifs were characterized, named Motif1–Motif14. In the conserved motif map, the relative degree of conservatism increased with the height of the letter and the frequency of the base corresponding to the letter. As shown in Figure 2c, OsAKT2 does not contain Motif7 and Motif9, while OsAKT1 and OsAKT3 contain 14 Motifs in the same number and order (Supplementary Table S3). The similarities and differences shown by these conserved motifs may be attributed to the adaptation of the functions of *OsShaker* K^+^ channel proteins to the evolutionary direction during the evolutionary process.

### 2.3. Phylogenetic and Gene Duplication Analysis of the OsShaker K^+^ Channel Genes

**Figure 3:**
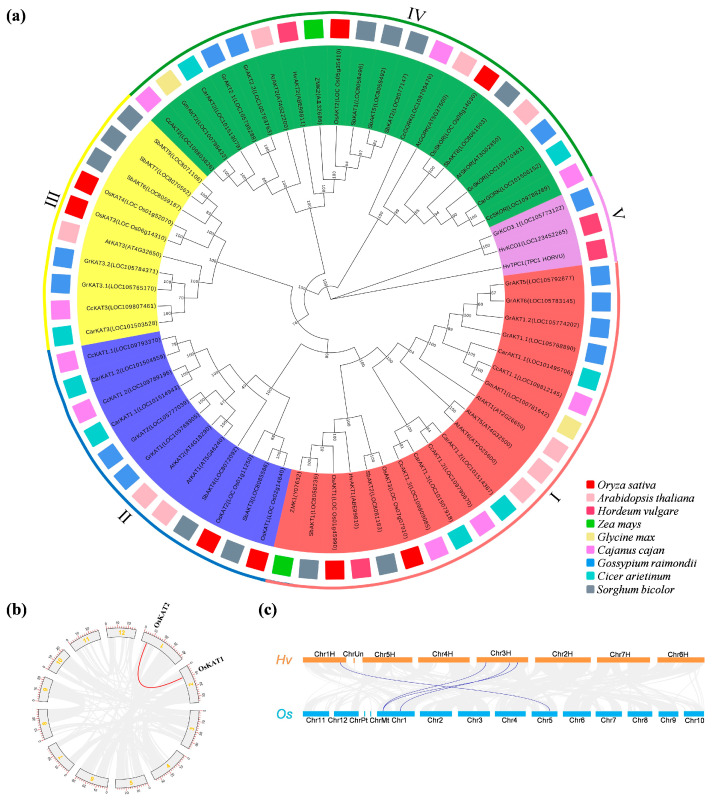
Phylogenetic, gene duplication and collinearity analysis of *OsShaker* K^+^ channel genes. (**a**) Phylogenetic analysis of the *Shaker* gene in *Oryza sativa*, *Arabidopsis thaliana*, *Hordeum vulgare*, *Zea mays*, *Glycine max*, *Cajanus cajan*, *Gossypium raimondii*, *Cicer arietinum*, *Sorghum bicolor*. *Oryza sativa*, *Arabidopsis thaliana*, *Hordeum vulgare*, *Zea mays*, *Glycine max*, *Cajanus cajan*, *Gossypium raimondii*, *Cicer arietinum*, *Sorghum bicolor* are prefixed with Os, At, Hv, Zm, Gm, Cc, Gr, Car and Sb, respectively. (**b**) Gene segmental duplications of the *OsShaker* K^+^ channel genes; the red curve represents one pair of segmental duplicated genes. The scale bar at the periphery of the chromosome represents the physical location (Kb). (**c**) Collinearity analysis of *OsShaker K^+^ genes* in rice and barley.

To further investigate the *OsShaker* K^+^ channel genes’ evolutionary relationship between rice and other species, such as *Arabidopsis thaliana*, *Hordeum vulgare*, *Zea mays*, and *Glycine max*, a phylogenetic tree was constructed. The results showed that *Shaker* K^+^ channel proteins are divided into five subfamilies (Clusters I–V)., and the proteins of *OsShaker* K^+^ channel were closely related to *Shaker* in monocotyledonous plants, such as barley and corn (Figure 3a), indicating that the *Shaker* K^+^ channel gene family in monocotyledonous and dicotyledonous plants shows different evolutionary trends.

Using TBtools, one pair of segmental duplication events was identified in the *OsShaker* K^+^ channel family, including *OsKAT2* on chromosome 1 and *OsKAT1* on chromosome 2 (Figure 3b). In addition, interspecies collinearity analysis revealed that there are bonds of *Shaker* K^+^ channel genes between rice and barley. Three *OsShaker* K^+^ channel family members (*OsAKT1*, *OsAKT2*, and *OsKAT2*) and four barley *Shaker* gene family members had covariate duplications (Figure 3c, Supplementary Table S4), indicating that the *OsShaker* family and the barley *Shaker* family are closely related during the evolutionary process.

### 2.4. Protein Structures and Transmembrane Domain Analysis of OsShaker K^+^ Channel Proteins

**Figure 4:**
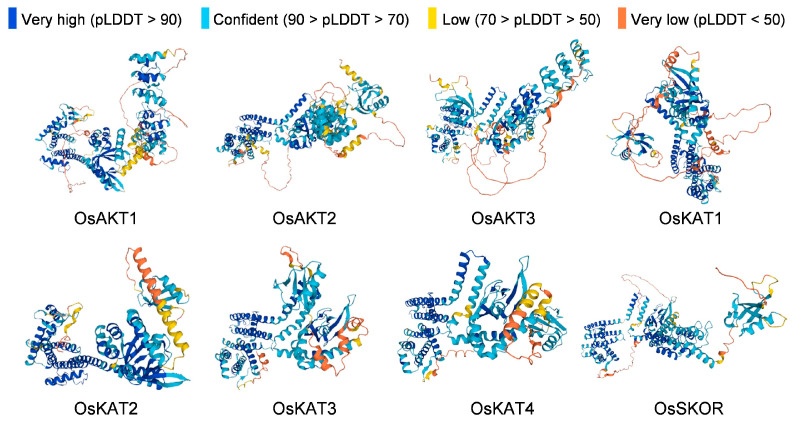
Protein 3D structure prediction model of *OsShaker* K^+^ channel proteins. AlphaFold produces a per-residue confidence score (pLDDT) between 0 and 100.

**Figure 5:**
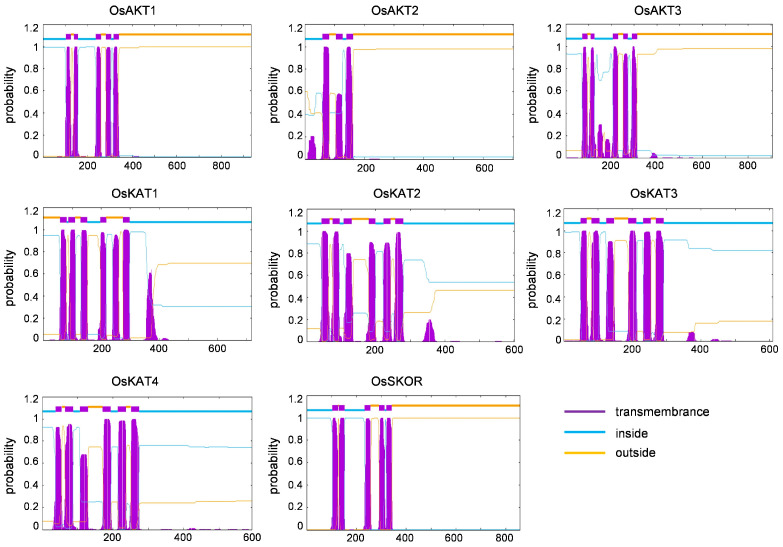
Transmembrane structure analysis of *OsShaker* K^+^ channel proteins.

Four secondary structures of *OsShaker* K^+^ channel proteins were characterized, i.e., alpha(α) helix, beta(β) turn, random coil and extended strand, and all were in the ratio of α-helix > random coil > extended strand > β-turn (Table 2). Accordingly, it can be noticed that α-helix and random coil are the main components of *OsShaker* K^+^ channel proteins, while β-turn and extended strand are scattered throughout the protein sequence, playing an auxiliary role in modification. The three-dimensional structure of *OsShaker* K^+^ channel proteins was analyzed, and the results are shown in Figure 4, indicating that the three-dimensional structures of *OsShaker* K^+^ channel proteins show a similarity. As shown in Figure 5, the transmembrane domain analysis of *OsShaker* K^+^ channel proteins showed that they all have transmembrane structural domains, and the predicted number of transmembrane helices is 3–6 (Figure 5, Supplementary Table S5). OsAKT2 contains three transmembrane domains, and OsKAT2, OsKAT3, OsKAT4 contain six transmembrane structural domains, while the remaining *OsShaker* K^+^ channel proteins have five transmembrane structural domains (Figure 5).

### 2.5. The Cis-Acting Element Analysis of OsShaker K^+^ Channel Genes Promoter

**Figure 6:**
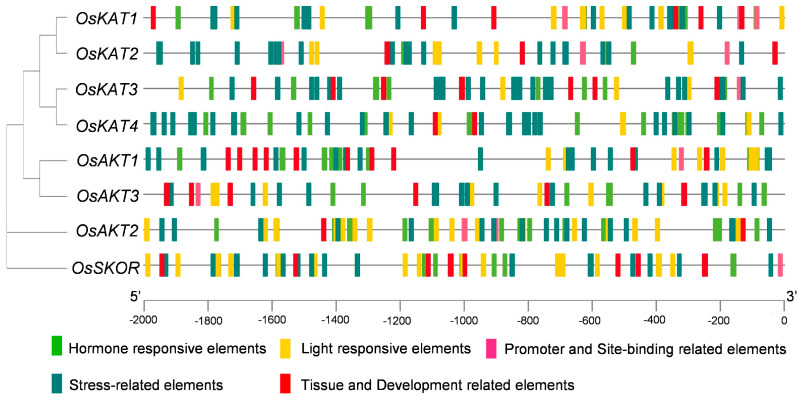
Prediction of cis-acting elements in *OsShaker* K^+^ channel genes promoter. Promoter sequences (−2 Kb) of *OsShaker* K^+^ channel genes were analyzed by PlantCARE. Different cis-elements are represented by different colors.

The cis-acting elements in the promoter play a critical role in the regulation of gene expression. The analysis of cis-acting elements of *OsShaker* K^+^ channel genes showed that the promoter sequences contained hormone-responsive cis-acting elements (eABRE, as-1, AuxRR-core, CGTCA-motif, ERE, GARE-motif, TATC-box, TGACG-box, TGACG-box, TGACG-box, TGACG-box, TGACG-box, TGACG-box, TGACG-box, TGACG-box, etc.), light-responsive cis-acting elements (AAGAA-motif, AE-box, Box4, G-Box, GATT-motif, ATC-motif, GT1-motif, Sp1, TCCC-motif, ACE, etc.), stress-responsive cis-acting elements (AP-1, ARE, DRE core, LTR, MYB, MYC, STRE, TC-rich repeats, W box, WRE3, etc.), promoter and site-binding-associated elements (CCAAT-box, MBS, MRE, CTAG-motif), and tissue and developmental elements (AAGAA-motif, ATC-motif, ATC-motif, ATC1-motif, Sp1, TCCC-motif, ACE, etc.) (Figure 6, Supplementary Table S6). The results indicate that *OsShaker* K^+^ channel genes may be regulated by hormones, light, and abiotic stresses. Among them, *OsAKT1* and *OsKAT2* contained AP-1 elements in response to cadmium stress, and *OsSKOR* contained LTR elements in response to stress. *OsKAT3* contained seven drought stress-responsive elements and *OsKAT4* contained eight cis-regulatory elements necessary for anaerobic induction.

### 2.6. OsShaker K^+^ Channel Proteins’ PPI Network Analysis

**Figure 7:**
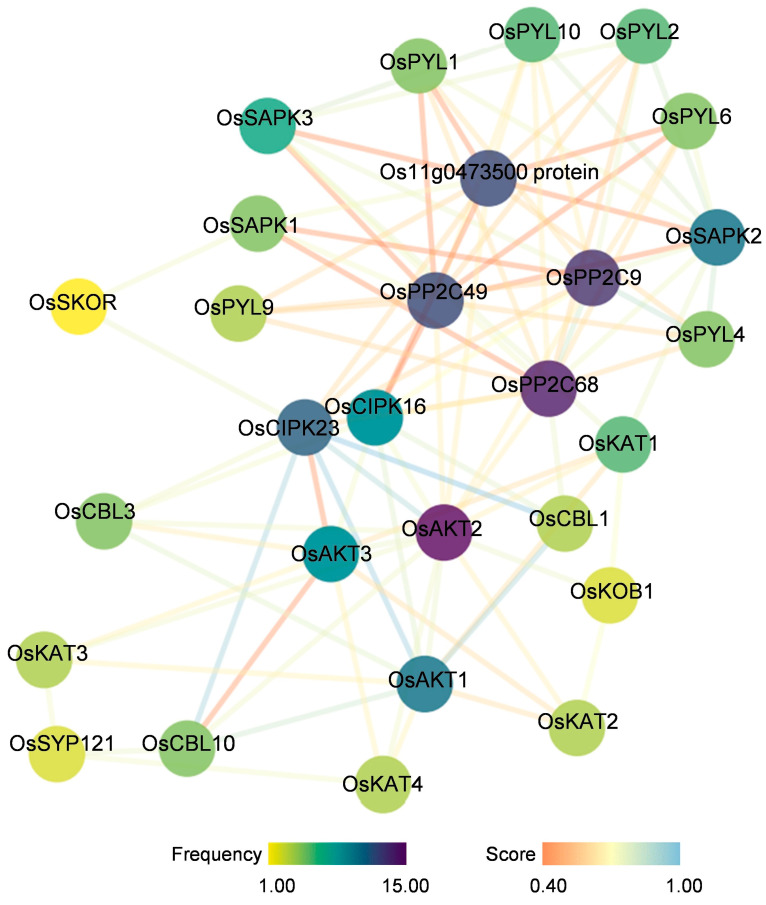
Protein–protein interaction (PPI) network analysis of *OsShaker* K^+^ channel proteins. Nodes represent proteins with direct interactions, and colors represent interaction frequencies; edges represent different types of interactions, and colors represent the strength of interactions.

The PPI network analysis of *OsShaker* K^+^ channel proteins was performed using the Interacting Protein Prediction STRING website and Cytoscape software. As shown in Figure 7, OsAKT1, OsAKT2, OsAKT3 and OsSKOR proteins potentially interacted with OsCIPK23, which is the calcium-regulated phosphatase class B protein. OsCBL1 and OsCIPK23 have been identified as upstream regulators of OsAKT1. OsAKT2 interacts with OsAKT1, OsAKT3, OsKAT1, OsKAT2, OsKAT3, and OsKAT4, whereas the OsSKOR only interacted with OsCIPK23 and OsSAPK1 proteins. The results indicate that *OsShaker* K^+^ channel proteins could perform different physiological functions by interacting with a variety of other proteins.

### 2.7. OsShaker K^+^ Channel Gene Expression Profiling and Tissue Specificity Analysis

**Figure 8:**
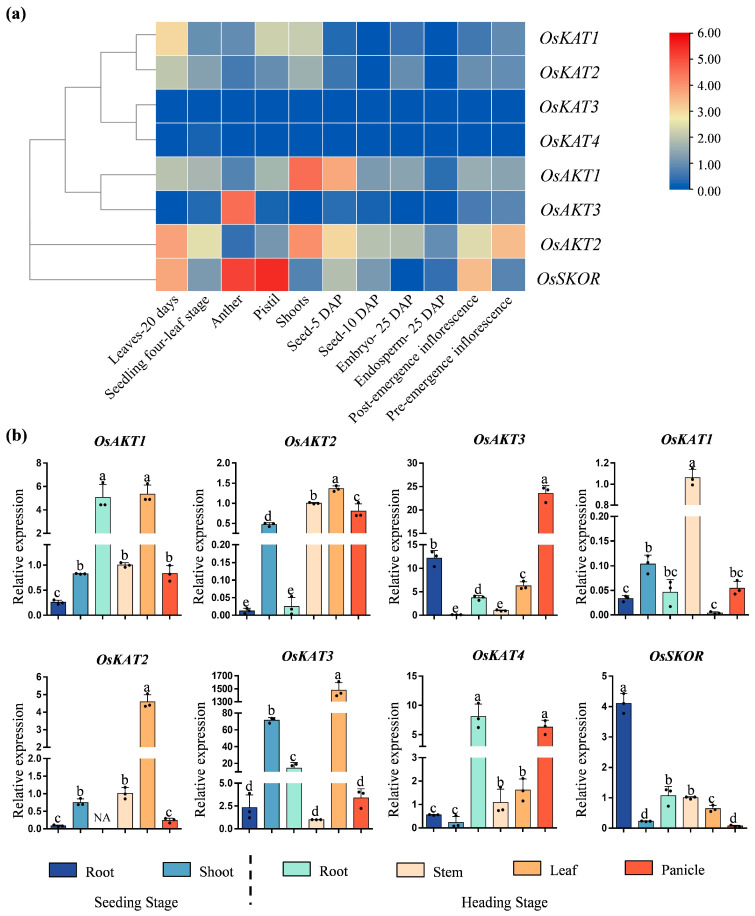
Tissue expression pattern of *OsShaker* K^+^ channel genes. (**a**) The expression profiles of the *OsShaker* K^+^ channel genes in the indica rice variety Minghui 63 obtained through the CREP database. The color scale represents relative expression levels from low (blue) to high (red). (**b**) The expression of *OsShaker* K^+^ channel genes in root, shoot, stem, leaf, and panicle. *OsUBQ5* was used for internal parameter, and transcript abundance was normalized to Stem. The data represent means ± standard deviation (*n* = 3); different letters indicate significant differences.

Hierarchical clustering of the expression of *OsShaker* K^+^ channel genes in different organs and developmental periods obtained from the database on the RGAP website showed that *OsSKOR* had the highest expression in stamens and anthers, whereas *OsAKT1* and *OsAKT2* were found to be predominantly expressed in the shoots. *OsKAT3* and *OsKAT4* were poorly expressed in all tissues and at all developmental stages of rice. Tissue-specific analyses of roots and shoots at seedling stage, as well as roots, stems, leaves at tasseling stage of ZH11, showed that the expression of *OsAKT3* and *OsSKOR* was higher in roots at seedling stage, and the expression of *OsAKT1*, *OsAKT2, OsKAT1,OsKAT2*, and *OsKAT3* were higher in shoots at seedling stage. In conclusion the expression of *OsShaker* K^+^ channel genes was spatio-temporally specific (Figure 8, Supplementary Table S7).

### 2.8. Expression Pattern of OsShaker K^+^ Channel Genes under Salt Stress

**Figure 9:**
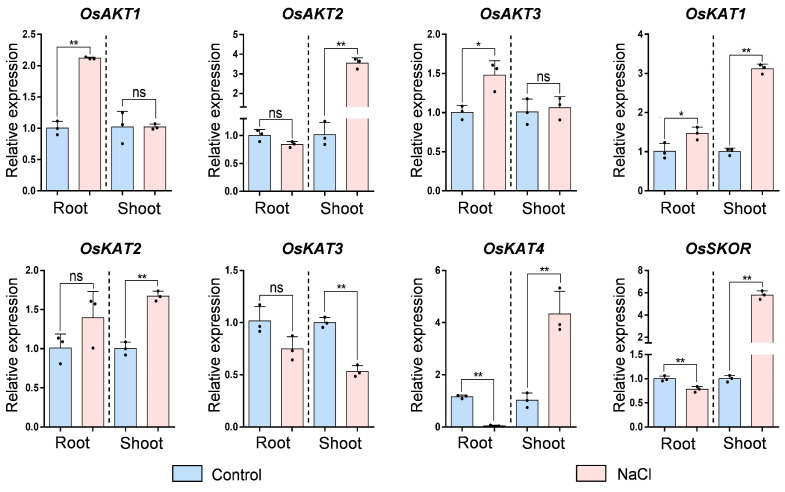
The expression analysis of *OsShaker* K^+^ channel genes under salt stress. *OsUBQ5* was used for normalization, and transcript abundance was normalized to Control. The data represent means ± standard deviation (*n* = 3). Asterisks represents significant differences from Control (* *p* < 0.05; ** *p* < 0.01) by Student’s *t*-test; ns indicates no significant difference.

In order to study the response of *OsShaker* K^+^ channel genes to salt stress, the expression of *OsShaker* K^+^ channel genes of rice seedlings was analyzed (Figure 9). In the root, *OsAKT1*, *OsAKT3*, *OsKAT1* and *OsKAT2* were up-regulated, while the expression level of *OsKAT4*, *OsSKOR* were down-regulated. The expression of *OsAKT1* was up-regulated by 2.11-fold compared with the control. In the shoot, the expression of *OsAKT2*, *OsKAT1*, *OsKAT2*, *OsKAT4* and *OsSKOR* was induced to be up-regulated, among which the expression of *OsSKOR* was up-regulated by 5.80-fold. In contrast, the *OsKAT3* expression was down-regulated by salt stress in shoot, with about 1.9-fold. In general, *OsShaker* K^+^ channel genes’ expression in response to salt stress suggests that *OsShaker* K^+^ channel genes may have different functions under salt stress.

### 2.9. Expression Pattern of OsShaker K^+^ Channel Genes under Chilling Treatment

**Figure 10:**
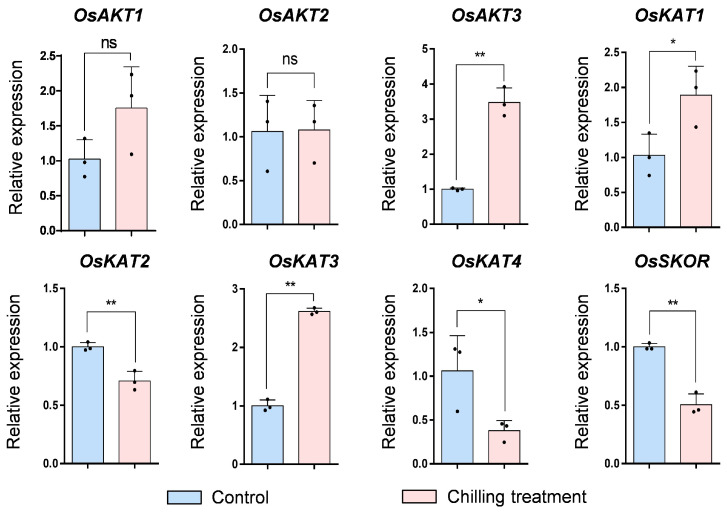
The expression analysis of *OsShaker* K^+^ channel genes with chilling treatment. The data represent means ± standard deviation (*n* = 3). *OsUBQ5* was used for normalization, and transcript abundance was normalized to Control. Asterisks represents significant differences from Control (* *p* < 0.05; ** *p* < 0.01) by Student’s *t*-test; ns indicates no significant difference.

To investigate the response of *OsShaker* K^+^ channel genes to low temperature stress, after 12 hours of chilling stress treatment, the expression level of *OsShaker* K^+^ channel genes in rice seedlings was analyzed by RT–qPCR. The results suggested that *OsAKT3*, *OsKAT1* and *OsKAT3* were induced, while *OsKAT2*, *OsKAT4*, *OsSKOR* were down-regulated in the shoot; however, *OsAKT1* and *OsAKT2* gene expression was not affected by cold stress. (Figure 10). Therefore, it can be concluded that *OsShaker* K^+^ channel genes may also play an important role in response to low temperature.

## 3. Discussion

Soil salinity severely inhibits rice production. The intracellular Na^+^/K^+^ ratio is a key factor in determining the salt tolerance of plants [35]. Potassium channels are responsible for the uptake and transport of Na^+^ and K^+^ in plants, among which the *Shaker* family is the earliest identified potassium channel, which is involved in the regulation of plant salt tolerance and development [36]. The *Shaker* family has been systematically identified in *Arabidopsis thaliana*, *Hordeum vulgare*, *Cajanus cajan*, *Gossypium raimondii*, etc. [31,37,38,39,40,41,42], but has been rarely studied in rice. In this study, 8 *OsShaker* K^+^ channel genes were identified in the rice genome.

Gene structure and conserved motifs influence their function. In this study, most of the *OsShaker* K^+^ channel genes were characterized as having multiple introns, but *OsKAT3* has no introns. Intronless genes are typically found in bacteria and primitive eukaryotes [43]. Thus, it is implied that *OsKAT3* is perhaps evolutionarily more conserved. In addition, the number and arrangement of motifs are the same in the same subfamily. For example, *OsAKT1* and *OsAKT3* have 15 motifs, and *OsAKT2* has only 7 motifs, suggesting that genes in the same subfamily may have similar functions. Repeatability and covariance analyses of the *OsShaker* K^+^ channel genes demonstrated OsKAT1 and OsKAT2, a pair of fragment repeat events, both of which are potassium inward rectifier channel proteins. The predicted subcellular localization results showed that the *OsShaker* K^+^ channel proteins were all localized in the cytoplasmic membrane, among which OsAKT2 was identified to be specifically localized in the plasma membrane [28,44].

Cis-acting elements in the promoter region are involved in the regulation of gene expression and can be used to predict biological functions, like stress response [45]. A variety of cis-acting elements, such as stress-responsive elements, light- and hormone-responsive elements, and tissue development-associated elements, were identified on the promoters of the *OsShaker* K^+^ channel genes, suggesting that the *OsShaker* K^+^ channel genes may be regulated by hormones, light, and adversity stresses, resulting in the regulation of rice growth, development, and response to adversity. Among them, *OsKAT3* contained seven drought stress-responsive elements, and *OsKAT4* contained eight cis-regulatory elements necessary for anaerobic induction. Analysis of interactions among *OsShaker* K^+^ channel proteins showed that OsAKT2 interacts with six *OsShaker* K^+^ channel members, except OsSKOR, which interacts with the ABA-responsive protein OsSAPK1. Moreover, the calmodulin phosphatase class B protein-interacting protein OsCIPK23, interacts with OsAKT1 [45]. In addition CIPK23 interactions with OsAKT1 have been experimentally demonstrated [46].

Potassium is one of the essential nutrients for plant growth and development, which can be taken up by plants from the environment through K^+^ channels or transporter proteins in the root [47]. Transmembrane structure analysis of *OsShaker* K^+^ channel proteins showed that they all have transmembrane structures, most of the proteins have 5–6 spans, and it is speculated that the *OsShaker* K^+^ channel proteins may have the function of mediating the transmembrane transport of potassium ions. In rice, overexpression of *OsAKT1* increases potassium ion uptake in roots, whereas the mutant of the *OsAKT1* plant showed a significant reduction in potassium ion content in vivo and exhibited a potassium-deficient phenotype [48]. *OsAKT2* possesses the ability to mediate K^+^ uptake in yeast and *Xenopus* oocytes [47], while AtAKT1 and AtHAK5 are responsible for K^+^ uptake in *Arabidopsis* [49].

*Shaker* K^+^ channel genes are widely involved in plant stomatal movement and drought stress response. In *Arabidopsis*, KAT1 and its homologous KAT2 are the primary inward rectifying channels in guard cells, mediating the influx of K^+^ into these cells, leading to stomatal opening [50]. However, GORK is the only outward-rectifying K^+^ channel in guard cells, which facilitate the efflux of K^+^, resulting in stomatal closure [51,52]. AtAKT1 and AtAKT2 are involved in the drought stress response, and the *AKT1* mutant exhibits increased drought tolerance [53]. Additionally, HvAKT1 and HvAKT2 have also been identified as being involved in the regulation of drought tolerance in barley [54]. Here, four *KAT*, three *AKT* and one *SKOR* have been identified in the rice genome, among which *OsAKT1* has been reported to be involved in drought regulation [48] Gene expression under stress and the site of gene expression are closely related to gene function [45]. Tissue expression of *OsShaker* K^+^ channel genes showed that *OsAKT2* and *OsKAT2* were mainly expressed in the shoot, whereas *OsAKT3* and *OsSKOR* were mainly expressed in the root. Among them, *OsAKT2* has been reported to mediate the distribution of K^+^ among different leaves [27,28]. The expression of *OsShaker* K^+^ channel genes was either up-regulated or down-regulated under salt stress, suggesting that these genes may be involved in the response process to salt stress in rice. The expression of *OsAKT1* was up-regulated in roots after salt stress treatment, as in the result of Golldack et al [55]. In this study, it was found that the expression of *OsAKT2* was significantly up-regulated in the shoot by salt stress, whereas the expression level in the roots was very low and no significant difference was obtained between normal condition and salt stress, which is consistent with the previous report [28]. Overexpression of *OsAKT2* improved salt tolerance in *Arabidopsis* by reabsorbing K^+^ in the phloem sap and leaves [27]. In addition, in rice, *OsAKT2* promotes the reabsorption of K^+^ from the phloem, controlling the distribution of shoot K^+^ among different leaves, as well as increasing the K^+^/Na^+^ ratio in young leaves and other growth points to enhance the salt tolerance under salt stress [28]. The different expression sites and the responding path to salt stress of the *OsShaker* K^+^ channel genes suggests that they may play different functions in different parts of the plant at different time points of the day during salt stress.

## 4. Materials and Methods

### 4.1. Gene Identification and Chromosomal Localization Analysis

The *Arabidopsis* Information Resource (TAIR) (http://www.arabidopsis.org/, accessed on 15 January 2024) was used to download the Arabidopsis *Shaker* protein sequences. The genome information of *OsShaker* K^+^ channel genes was searched for in the InterPro database, based on the reported *Arabidopsis Shaker* family members. SMART(http://smart.embl-heidelberg.de/, accessed on 16 January 2024),an online tool, was used to identify the relevant domains (Ion_trans_2 structural domains, cNMP structural domains, ANK structural domains and KHA structural domains). *OsShaker* K^+^ channel protein information wsd collected from Rice Date (https://www.ricedata.cn/gene/, accessed on 17 January 2024) and the Rice Genome Annotation Project (http://rice.plantbiology.msu.edu/index.shtml), and utilized TBtools II v2.031 [56] to create chromosomal localization maps.

### 4.2. Protein Sequence Comparison, Phylogenetic Tree and Gene Duplication Analysis

Sequence comparison and output of the conserved structural domains of *OsShaker* K^+^ channel genes were accomplished by MEGA7.0 and geneDoc. The phylogenetic trees of rice, *Arabidopsis*, barley, soybean, and corn *Shaker K^+^ channel* gene family were constructed in MEGA7.0 using the neighbor-joining method., and their amino acid sequences were download from the Ensembl Plants database (https://plants.ensembl.org/index.html, accessed on 22 January 2024) [57]. Analyses with 1000 replicates were conducted to evaluate the phylogenetic tree. The segmental duplication events of *Shaker K^+^ channel* gene family proteins between rice and barley were analyzed using TBtools II v2.031. An all-vs-all BLASTP local search was performed among rice and barley to identify potential homologous gene pairs (E < 1 × 10^−10^). With homologous pairs as input, homologous chains were identified by MCScanX software. The results were presented in the form of Circos plots [56].

### 4.3. Gene Structure and Conserved Motif Analysis

The coding sequences (CDS) and their corresponding genomic DNA sequences of *OsShaker K^+^* channel genes were downloaded in FASTA format on the Ensembl Plants database (http://plants.ensembl.org/index.html, accessed on 2 February 2024) [57]. Search for the specified genotype number on the Home and click ‘Export data’, and then select GFF3 (Generic Feature Format Version 3) format Output. The distribution of introns and exons and non-coding regions of the genes was mapped using TBtools II v2.031. The online tool MEME (http://meme-suite.org/, accessed on 2 February 2024) was used to search for motifs (we found the most statistically significant (low E-value, E-value < 0.05) motifs), and the conserved motifs were beautified using TBtools II v2.031 [56].

### 4.4. Protein Physicochemical Properties and Subcellular Localization Analysis

Protein physicochemical properties were analyzed by ExPASy (https://web.expasy.org/protparam/, accessed on 23 January 2024). We perform the compute parameters after keying the protein sequence, then find the corresponding calculation result in the output interface. In addition, we made predictions about subcellular localization through the WoLF (https://wolfpsort.hgc.jp/, accessed on 23 January 2024), and it is important to note here that we first selected the plant option. Nuclear localization sequence analysis by NucPred (https://nucpred.bioinfo.se/nucpred/, accessed on 23 January 2024). The NucPred score was greater than 0.6, the nucleated localization sequence was indicated as “Positive”.

### 4.5. Protein Structures and Transmembrane Structural Domain Analysis

The secondary and tertiary structures of the protein were analyzed through the SOPMA (https://npsa-prabi.ibcp.fr/cgi-bin/npsa_automat.pl?page=/NPSA/npsa_sopma_f.html, accessed on 12 February 2024) and the SWISS-MODEL (https://swissmodel.expasy.org/, accessed on 13 February 2024), respectively. Among them, the SOPMA website needs to enter the protein sequence, and the Swiss-model website can enter the protein sequence or protein abbreviation. The protein transmembrane structural domains were analysed by the online software tool TMHMM 2.0 (https://services.healthtech.dtu.dk/service.php?DeepTMHMM, accessed on 13 February 2024) [58]. It should be noted here that, after the input of the protein sequence, the following output format has to be selected: ‘Extensive, with graphics’.

### 4.6. Cis-Acting Elements Analysis

Download 2000 bp upstream of the *OsShaker* K^+^ channel genes on the Ensembl (http://plants.ensembl.org/index.html, accessed on 27 January 2024) as the promoter sequence. Select ‘FASTA sequence’ format Output in ‘Export data’. The cis-acting elements predictions of the promoter were performed by PlantCare (http://bioinformatics.psb.ugent.be/webtools/plantcare/html/, accessed on 27 January 2024). After receiving the email from PlantCare, download the file in tab format for preliminary statistical analysis. Then the promoter cis-acting elements were mapped by TBtools II v2.031 software [56,59].

### 4.7. Plant Growth and Stress Treatments

The *Oryza sativa* cv. *japonica* cultivar Zhonghua 11 (ZH11) used in this study was obtained from the China National Rice Research Institute (Hangzhou, China). Seedlings were grown in an artificial climate chamber(Intelligent light incubator #GXM-508C-4, Ningbo Jiangnan Instrument Factory, China) under the following conditions: 14 h of light at 28 °C, 10 h of dark at 24 °C, 70% humidity, and a light intensity 18,000 lx.

Salt treatment: Two-week-old hydroponic seedlings were transferred to the nutrient solution with 140 mM NaCl for treatment, and the shoot and root were taken for gene expression analysis after 5 h of salt stress [28].

Chilling treatment: Two-week-old hydroponic seedlings were treated under cold stress (4 °C). The shoots of the seedlings were collected after 12 h of treatment for RT–qPCR [60].

### 4.8. RNA Extraction and RT–qPCR Analysis

The total RNA was extracted by using Plant Total RNA Kit (Tiangen Biochemical Technology Co., Ltd. Code: DP432, Beijing, China), and 1 μg RNA was reversed transcribed into cDNA by using HiScriptIII 1st Strand cDNA Synthesis Kit (Vazyme Code: R312-02). PCR amplification was performed using a CFX96 Real-Time PCR Detection System (Bio-Rad, Hercules, CA, USA). The reaction conditions were pre-denaturation at 95 °C for 5 min, denaturation at 94 °C for 30 s, annealing at 60 °C for 40 s, and extension at 72 °C for 20 s. Forty amplification cycles were performed, with three replicates, and the data were processed by 2^−ΔΔCt^ [61,62]. Primer Premier 5 was used to design primers for RT–qPCR, and *OsUBQ5* was selected as the internal reference gene.

### 4.9. Statistical Analysis

Microsoft Excel 2010 and GraphPad Prism 8.0 were used for data analysis and fig. construction. The general linear model procedure in SPSS 21.0 was used for analysis of variance (ANOVA). Mean values were compared using Duncan’s multiple comparison procedure at the 1% or 5% level of probability and the single-sample *t*-test (* *p* < 0.05; ** *p* < 0.01).

## 5. Conclusions

In this study, genome-wide identification and expression pattern analysis of *OsShaker* K^+^ channel genes were performed in rice. Eight *OsShaker* K^+^ channel genes were identified in the rice genome and divided into five groups. Members of the same subfamily have similar genetic characteristics. All *OsShaker* K^+^ channel proteins were predicted to localized in the cytoplasmic membrane. The promoters of *OsShaker* K^+^ channel gene contain certain elements which respond to abiotic stresses. In addition, the RT–qPCR analysis suggested that the expression of *OsShaker* K^+^ channel gene was significantly influenced by salt and chilling. According to the findings, it can be concluded that *OsShaker* K^+^ channel genes may play an important role in salt stress and chilling stress response. Overall, the results of this study enriched the understanding of the *Shaker* K^+^ channel family, providing a theoretical basis for in-depth research on their functions in the response to abiotic stress, as well as the cultivation of new abiotic-tolerant rice varieties.

## Figures and Tables

**Table 1:** Physiochemical properties of the *OsShaker* K^+^ channel genes.

Name	Gene ID	Accession Number	Chromosome Position	Length (aa)	MW (kDa)	PI	GRAVY	Localization	NLS Predicts
*OsAKT1*	*Os01g0648000*	*LOC_Os01g45990*	Chr1	935	102.02	6.78	−0.149	Plas	NA
*OsAKT2*	*Os05g0428700*	*LOC_Os05g35410*	Chr5	703	76.70	6.40	−0.071	Plas	NA
*OsAKT3*	*Os07g0175400*	*LOC_Os07g07910*	Chr7	711	77.46	10.9	−0.355	Plas	Positive
*OsKAT1*	*Os02g0245800*	*LOC_Os02g14840*	Chr2	718	81.03	6.76	−0.243	Plas	Positive
*OsKAT2*	*Os01g0210700*	*LOC_Os01g11250*	Chr1	601	68.17	8.57	−0.076	Plas	NA
*OsKAT3*	*Os06g0254200*	*LOC_Os06g14310*	Chr6	610	67.73	9.03	0.149	Plas	NA
*OsKAT4*	*Os01g0718700*	*LOC_Os01g52070*	Chr1	373	41.67	9.19	0.308	Plas	NA
*OsSKOR*	*Os06g0250600*	*LOC_Os06g14030*	Chr6	858	95.10	5.66	−0.211	Plas	NA

Note: MW, Molecular weight (kDa); pI, Isoelectric point; GRAVY, Grand average of hydropathicity; NLS predicts, Nuclear Localization Signal predicts. Plas, Plasma membrane. “NA” is an abbreviation for “Not Available” and indicates no NLS. “Positive” indicates the presence of NLS.

**Table 2:** Secondary structure analysis of *OsShaker* K^+^ channel proteins.

Protein Name	Percentage				Distribution of Secondary Structure Elements
	Ah 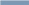	Bt 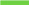	Rc 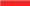	Es 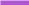	
OsAKT1	40.43	7.38	37.86	14.33	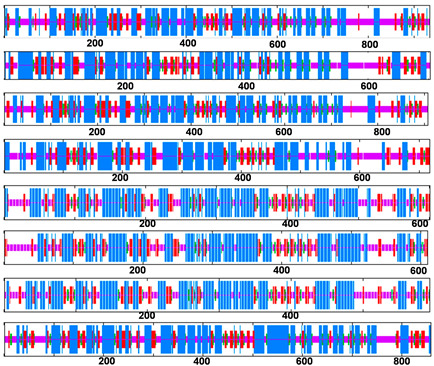
OsAKT2	43.10	7.11	33.71	16.07	
OsAKT3	40.79	7.50	35.50	16.21	
OsKAT1	41.36	4.32	38.02	16.30	
OsKAT2	47.92	4.99	28.79	18.30	
OsKAT3	46.39	4.75	30.00	18.85	
OsKAT4	45.36	5.90	29.51	19.22	
OsSKOR	44.76	8.28	30.89	16.08	

Note: In the secondary structure component distribution diagram, the blue structure represents Alpha helix (Ah), the green structure represents Beta turn (Bt), the yellow structure represents Random coil (Rc), and the red structure represents Extended strand (Es).

## Data Availability

Data are contained within the article and Supplementary Materials.

## Supplementary Materials

The following supporting information can be downloaded at: https://www.mdpi.com/article/10.3390/ijms25179728/s1.
